# *catena*-Poly[[[2-(1,3-thia­zol-4-yl)-1*H*-benzimidazole]­manganese(II)]-μ-oxalato]

**DOI:** 10.1107/S1600536813023428

**Published:** 2013-08-23

**Authors:** Peng Liang, Li-Ning Huang, Xian-Hong Yin

**Affiliations:** a College of Chemistry and Chemical Engineering, Guangxi University for Nationalities, Nanning 530006, People’s Republic of China; b Nonferrous Geological Prospecting Bureau, Hunan Non-ferrous Geology Exploration and Institute, Changsha 410015, People’s Republic of China

## Abstract

In the title compound, [Mn(C_2_O_4_)(C_10_H_7_N_3_S)]_*n*_, the Mn^II^ cation is chelated by one 2-(1,3-thia­zol-4-yl)-1*H*-benzimidazole ligand and two oxalate anions in a distorted N_2_O_4_ octa­hedral geometry. Two independent oxalate anions are located on individual inversion centers and bridge the Mn^II^ cations into a polymeric chain running along [101]. The thia­zole ring is approximately coplanar with the benzimidazole ring system [dihedral angle = 4.19 (9)°]. In the crystal, classical N—H⋯O hydrogen bonds and weak C—H⋯O hydrogen bonds link the polymeric chains into a three-dimensional supra­molecular architecture.

## Related literature

For applications of thia­bendazole compounds, see: Yu *et al.* (2002 ▶); Devereux *et al.* (2004 ▶). For related structures, see: Wisniewski *et al.* (2001 ▶); Jean *et al.* (2002 ▶).
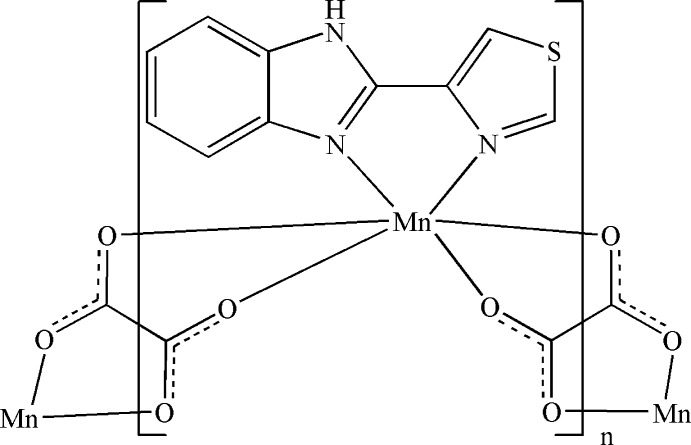


## Experimental

### 

#### Crystal data

[Mn(C_2_O_4_)(C_10_H_7_N_3_S)]
*M* *_r_* = 344.21
Monoclinic, 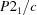

*a* = 9.374 (2) Å
*b* = 17.834 (5) Å
*c* = 8.926 (2) Å
β = 113.500 (3)°
*V* = 1368.5 (6) Å^3^
*Z* = 4
Mo *K*α radiation
μ = 1.14 mm^−1^
*T* = 296 K
0.19 × 0.15 × 0.12 mm

#### Data collection

Bruker SMART 1000 diffractometer
Absorption correction: multi-scan (*SADABS*; Bruker, 2001 ▶) *T*_min_ = 0.813, *T*_max_ = 0.876
7273 measured reflections
2412 independent reflections
2221 reflections with *I* > 2σ(*I*)
*R*_int_ = 0.017

#### Refinement

*R*[*F* ^2^ > 2σ(*F* ^2^)] = 0.024
*wR*(*F* ^2^) = 0.069
*S* = 1.08
2412 reflections
190 parameters
H-atom parameters constrained
Δρ_max_ = 0.27 e Å^−3^
Δρ_min_ = −0.28 e Å^−3^

### 

Data collection: *SMART* (Bruker, 2007 ▶); cell refinement: *SAINT* (Bruker, 2007 ▶); data reduction: *SAINT*; program(s) used to solve structure: *SHELXTL* (Sheldrick, 2008 ▶); program(s) used to refine structure: *SHELXTL*; molecular graphics: *SHELXTL*; software used to prepare material for publication: *SHELXTL*.

## Supplementary Material

Crystal structure: contains datablock(s) I, global. DOI: 10.1107/S1600536813023428/xu5729sup1.cif

Structure factors: contains datablock(s) I. DOI: 10.1107/S1600536813023428/xu5729Isup2.hkl

Supplementary material file. DOI: 10.1107/S1600536813023428/xu5729Isup3.cdx

Additional supplementary materials:  crystallographic information; 3D view; checkCIF report

## Figures and Tables

**Table 1:** Selected bond lengths (Å)

Mn1—O1	2.2146 (15)
Mn1—O2	2.2100 (14)
Mn1—O3	2.1640 (14)
Mn1—O4	2.1667 (15)
Mn1—N1	2.3170 (17)
Mn1—N2	2.2279 (16)

**Table 2:** Hydrogen-bond geometry (Å, °)

*D*—H⋯*A*	*D*—H	H⋯*A*	*D*⋯*A*	*D*—H⋯*A*
N3—H3⋯O1^i^	0.86	1.96	2.812 (2)	170
C12—H12⋯O3^ii^	0.93	2.52	3.137 (3)	124

Symmetry codes: (i) 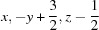; (ii) 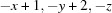.

**Fig. 1.**
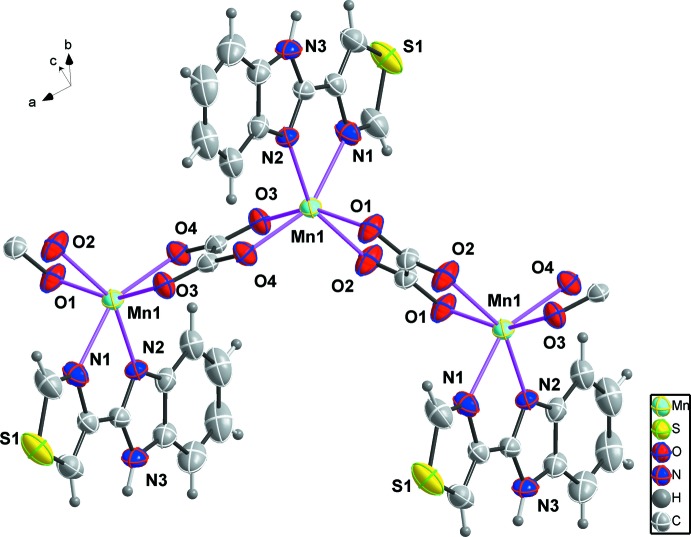
The asymmetric unit of the title compound. Displacement ellipsoids are drawn at the 30% probability level.

**Fig. 2.**
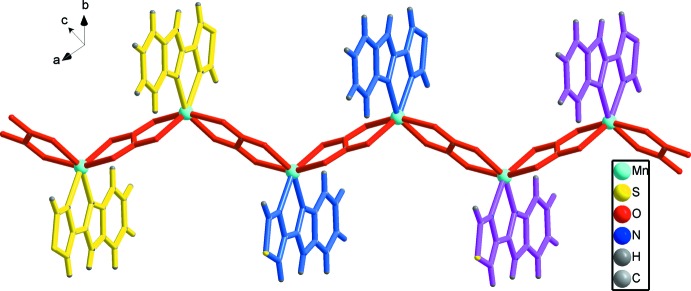
Perspective view of the chains in the title compound.

**Fig. 3.**
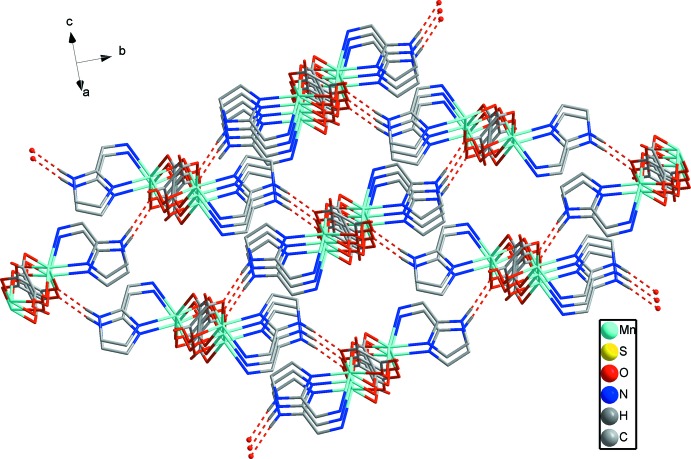
Crystal packing of the title compound. Dashed lines denote hydrogen bonds.

## supplementary crystallographic information

### 1. Comment

Thiabendazole aroused considerable interest in biology and medicine due to its antiproliferative activities. It is an antimicrobial drug belonging to the benzimidazole derivative, and has exhibited wide applications in human and veterinary medicine (Jean *et al.*, 2002; Devereux *et al.*, 2004).

As part of our studies of the synthesis and characterization of these compounds, we report here the synthesis and crystal structure of [Mn(C_2_O_4_)(thiabendazole)]_n_. In this work, the structure of the complex is formed by infinite one-dimensional chains. Each Mn(II) center is six-coordinated by two N-atoms (N1, N2) and four O-atoms (O1, O2, O3, O4) of the carboxylate from two H_2_C_2_O_4_ ligands and two N-atoms from a chelated tbz ligand (Fig. 1). The dihedral angle between the least squares calculated planes through the adjacent tbz (benzene ring) ligand is close to 90 °. The Mn—O bond lengths of 2.164–2.215 Å are shorter than the the Zn—N bond length of 2.228–2.317 Å, where Jahn-Teller effects have not been observed. The complex form a one-dimensional chain structure by bis(bidentate) bridging H_2_C_2_O_4_, and thiabendazole located on both sides of the chain (Fig. 2). The complex is stabilized by hydrogen bonds formed by N3—H3···O1 hydrogen bonds from N—H of tbz together with oxygen atoms of [C_2_O_4_]^2-^ ligands, their length is 1.962 Å and within the normal range. Because the direction of the hydrogen bonds is not the same, the hydrogen bonds interlink the 1-D chains to generate three-dimensional supramolecular architectures (Fig. 3) (Wisniewski *et al.*, 2001; Yu *et al.*, 2002).

### 2. Experimental

A solution of thiabendazole (0.2023 g, 1 mmol) in 5 ml DMF was added dropwise with stirring at room temperature to a solution of Mn(Cl)_2_.4H_2_O (0.1976 g, 1 mmol), H_2_C_2_O_4_ (0.0411 g, 0.5 mmol) in the mixture of 12.5 ml water and 5 ml methanol. Then an aqueous solution of sodium hydroxide was added dropwise with stirring to adjust the pH value of the solution being 6.5. The resulting mixture was sealed in a 25 mL Teflon-lined stainless reactor, kept under autogenous pressure at 423 K for 72 h, and then slowly cooled to room temperature at a rate of 10 K per hour. The colorless block crystals suitable for X-ray diffraction were isolated directly, washed with ethanol and dried in air (0.182 g, Yield: 41.2%, based on Mn). Elemental analysis calculate(%): C,41.87; H, 2.05; N,12.21. Elemental analysis: found(%): C,41.65; H,2.11; N,12.24.

### 3. Refinement

H atoms were positioned geometrically and refined as riding atoms with C—H = 0.93 and N—H = 0.86 Å, *U*_iso_(H) = 1.2*U*_eq_(C,N).

### Crystal data

**Table:**

[Mn(C_2_O_4_)(C_10_H_7_N_3_S)]	*F*(000) = 692
*M**_r_* = 344.21	*D*_x_ = 1.671 Mg m^−^^3^
Monoclinic, *P*2_1_/*c*	Mo *K*α radiation, λ = 0.71073 Å
Hall symbol: -P 2ybc	Cell parameters from 5023 reflections
*a* = 9.374 (2) Å	θ = 2.4–28.3°
*b* = 17.834 (5) Å	µ = 1.14 mm^−^^1^
*c* = 8.926 (2) Å	*T* = 296 K
β = 113.500 (3)°	Block, colorless
*V* = 1368.5 (6) Å^3^	0.19 × 0.15 × 0.12 mm
*Z* = 4	

### Data collection

**Table:**

Bruker SMART 1000 diffractometer	2412 independent reflections
Radiation source: fine-focus sealed tube	2221 reflections with *I* > 2σ(*I*)
Graphite monochromator	*R*_int_ = 0.017
phi and ω scans	θ_max_ = 25.0°, θ_min_ = 2.3°
Absorption correction: multi-scan (*SADABS*; Bruker, 2001)	*h* = −7→11
*T*_min_ = 0.813, *T*_max_ = 0.876	*k* = −21→20
7273 measured reflections	*l* = −10→10

### Refinement

**Table:**

Refinement on *F*^2^	Primary atom site location: structure-invariant direct methods
Least-squares matrix: full	Secondary atom site location: difference Fourier map
*R*[*F*^2^ > 2σ(*F*^2^)] = 0.024	Hydrogen site location: inferred from neighbouring sites
*wR*(*F*^2^) = 0.069	H-atom parameters constrained
*S* = 1.08	*w* = 1/[σ^2^(*F*_o_^2^) + (0.0339*P*)^2^ + 0.5684*P*] where *P* = (*F*_o_^2^ + 2*F*_c_^2^)/3
2412 reflections	(Δ/σ)_max_ = 0.001
190 parameters	Δρ_max_ = 0.27 e Å^−^^3^
0 restraints	Δρ_min_ = −0.28 e Å^−^^3^

### Special details

**Table:**

Geometry. All e.s.d.'s (except the e.s.d. in the dihedral angle between two l.s. planes) are estimated using the full covariance matrix. The cell e.s.d.'s are taken into account individually in the estimation of e.s.d.'s in distances, angles and torsion angles; correlations between e.s.d.'s in cell parameters are only used when they are defined by crystal symmetry. An approximate (isotropic) treatment of cell e.s.d.'s is used for estimating e.s.d.'s involving l.s. planes.
Refinement. Refinement of *F*^2^ against ALL reflections. The weighted *R*-factor *wR* and goodness of fit *S* are based on *F*^2^, conventional *R*-factors *R* are based on *F*, with *F* set to zero for negative *F*^2^. The threshold expression of *F*^2^ > σ(*F*^2^) is used only for calculating *R*-factors(gt) *etc*. and is not relevant to the choice of reflections for refinement. *R*-factors based on *F*^2^ are statistically about twice as large as those based on *F*, and *R*- factors based on ALL data will be even larger.

### Fractional atomic coordinates and isotropic or equivalent isotropic displacement parameters (Å^2^)

**Table:**

	*x*	*y*	*z*	*U*_iso_*/*U*_eq_	
Mn1	0.28290 (3)	0.932763 (14)	0.20300 (3)	0.03109 (11)	
S1	0.64896 (7)	0.83770 (4)	−0.00986 (9)	0.0624 (2)	
O1	0.39555 (17)	0.91664 (7)	0.47096 (16)	0.0433 (4)	
O2	0.43698 (18)	1.03068 (8)	0.29932 (16)	0.0484 (4)	
O3	0.17776 (14)	0.99693 (8)	−0.02014 (16)	0.0376 (3)	
O4	0.05151 (15)	0.95656 (8)	0.18979 (16)	0.0377 (3)	
N1	0.45900 (19)	0.87876 (9)	0.1125 (2)	0.0413 (4)	
N2	0.22949 (18)	0.81054 (8)	0.17676 (18)	0.0320 (3)	
N3	0.2776 (2)	0.69534 (9)	0.1121 (2)	0.0397 (4)	
H3	0.3214	0.6596	0.0814	0.048*	
C1	0.0364 (2)	1.01176 (9)	−0.0610 (2)	0.0304 (4)	
C2	0.3161 (2)	0.76883 (10)	0.1233 (2)	0.0322 (4)	
C3	0.1257 (2)	0.76095 (10)	0.1997 (2)	0.0345 (4)	
C4	0.4377 (2)	0.80249 (11)	0.0832 (2)	0.0356 (4)	
C5	0.4889 (2)	0.96704 (10)	0.5512 (2)	0.0336 (4)	
C6	0.1543 (2)	0.68852 (11)	0.1592 (2)	0.0408 (5)	
C7	0.0076 (2)	0.77466 (13)	0.2539 (3)	0.0445 (5)	
H7	−0.0116	0.8225	0.2831	0.053*	
C8	0.0644 (3)	0.62731 (13)	0.1659 (3)	0.0615 (7)	
H8	0.0825	0.5792	0.1372	0.074*	
C9	0.5322 (3)	0.77105 (13)	0.0185 (3)	0.0480 (5)	
H9	0.5331	0.7205	−0.0067	0.058*	
C10	−0.0793 (3)	0.71361 (15)	0.2620 (3)	0.0602 (7)	
H10	−0.1583	0.7206	0.2986	0.072*	
C11	−0.0529 (3)	0.64235 (16)	0.2177 (4)	0.0697 (8)	
H11	−0.1164	0.6033	0.2230	0.084*	
C12	0.5678 (3)	0.90352 (14)	0.0683 (3)	0.0537 (6)	
H12	0.5982	0.9535	0.0791	0.064*	

### Atomic displacement parameters (Å^2^)

**Table:**

	*U*^11^	*U*^22^	*U*^33^	*U*^12^	*U*^13^	*U*^23^
Mn1	0.02973 (17)	0.02287 (16)	0.03696 (18)	−0.00105 (10)	0.00938 (13)	−0.00085 (10)
S1	0.0522 (4)	0.0722 (4)	0.0788 (4)	0.0189 (3)	0.0429 (3)	0.0219 (3)
O1	0.0519 (9)	0.0291 (7)	0.0401 (7)	−0.0171 (6)	0.0090 (6)	0.0016 (6)
O2	0.0616 (9)	0.0343 (8)	0.0363 (8)	−0.0179 (7)	0.0058 (7)	0.0050 (6)
O3	0.0294 (7)	0.0425 (8)	0.0417 (7)	0.0018 (6)	0.0150 (6)	0.0054 (6)
O4	0.0372 (7)	0.0387 (7)	0.0376 (7)	0.0065 (6)	0.0152 (6)	0.0074 (6)
N1	0.0368 (9)	0.0326 (9)	0.0584 (11)	0.0025 (7)	0.0230 (8)	0.0052 (8)
N2	0.0325 (8)	0.0247 (8)	0.0367 (8)	−0.0019 (6)	0.0115 (6)	−0.0021 (6)
N3	0.0472 (10)	0.0238 (8)	0.0445 (9)	0.0038 (7)	0.0144 (8)	−0.0033 (7)
C1	0.0323 (10)	0.0224 (8)	0.0359 (10)	−0.0023 (7)	0.0130 (8)	−0.0040 (7)
C2	0.0340 (10)	0.0263 (9)	0.0327 (9)	0.0030 (7)	0.0095 (8)	−0.0013 (7)
C3	0.0330 (10)	0.0316 (10)	0.0335 (9)	−0.0042 (8)	0.0074 (8)	0.0026 (7)
C4	0.0340 (10)	0.0345 (10)	0.0357 (10)	0.0069 (8)	0.0112 (8)	0.0025 (8)
C5	0.0364 (10)	0.0219 (9)	0.0385 (10)	−0.0045 (7)	0.0106 (8)	0.0016 (8)
C6	0.0419 (11)	0.0293 (10)	0.0425 (11)	−0.0030 (8)	0.0077 (9)	0.0046 (8)
C7	0.0372 (11)	0.0505 (13)	0.0446 (11)	0.0007 (9)	0.0149 (9)	0.0096 (9)
C8	0.0638 (16)	0.0319 (12)	0.0713 (16)	−0.0098 (11)	0.0086 (13)	0.0109 (11)
C9	0.0470 (12)	0.0478 (13)	0.0505 (12)	0.0133 (10)	0.0208 (10)	0.0022 (10)
C10	0.0439 (13)	0.0677 (17)	0.0672 (15)	−0.0024 (12)	0.0203 (11)	0.0300 (13)
C11	0.0503 (15)	0.0589 (17)	0.0879 (19)	−0.0153 (12)	0.0149 (14)	0.0341 (14)
C12	0.0404 (12)	0.0462 (12)	0.0781 (16)	0.0069 (10)	0.0276 (11)	0.0182 (12)

### Geometric parameters (Å, º)

**Table:**

Mn1—O1	2.2146 (15)	C1—O4^ii^	1.251 (2)
Mn1—O2	2.2100 (14)	C1—C1^ii^	1.556 (4)
Mn1—O3	2.1640 (14)	C2—C4	1.453 (3)
Mn1—O4	2.1667 (15)	C3—C6	1.396 (3)
Mn1—N1	2.3170 (17)	C3—C7	1.396 (3)
Mn1—N2	2.2279 (16)	C4—C9	1.357 (3)
S1—C12	1.694 (3)	C5—O2^i^	1.235 (2)
S1—C9	1.702 (2)	C5—C5^i^	1.553 (3)
O1—C5	1.260 (2)	C6—C8	1.395 (3)
O2—C5^i^	1.235 (2)	C7—C10	1.378 (3)
O3—C1	1.254 (2)	C7—H7	0.9300
O4—C1^ii^	1.251 (2)	C8—C11	1.378 (4)
N1—C12	1.308 (3)	C8—H8	0.9300
N1—C4	1.384 (3)	C9—H9	0.9300
N2—C2	1.323 (2)	C10—C11	1.382 (4)
N2—C3	1.389 (2)	C10—H10	0.9300
N3—C2	1.353 (2)	C11—H11	0.9300
N3—C6	1.384 (3)	C12—H12	0.9300
N3—H3	0.8600		
			
O3—Mn1—O4	76.57 (5)	N2—C2—C4	120.73 (16)
O3—Mn1—O2	85.83 (5)	N3—C2—C4	126.47 (17)
O4—Mn1—O2	110.56 (6)	N2—C3—C6	109.46 (17)
O3—Mn1—O1	155.34 (5)	N2—C3—C7	129.70 (18)
O4—Mn1—O1	96.90 (5)	C6—C3—C7	120.84 (19)
O2—Mn1—O1	74.11 (5)	C9—C4—N1	114.63 (19)
O3—Mn1—N2	114.83 (5)	C9—C4—C2	130.04 (19)
O4—Mn1—N2	90.45 (6)	N1—C4—C2	115.32 (16)
O2—Mn1—N2	153.97 (6)	O2^i^—C5—O1	127.00 (17)
O1—Mn1—N2	88.68 (5)	O2^i^—C5—C5^i^	117.4 (2)
O3—Mn1—N1	91.44 (6)	O1—C5—C5^i^	115.6 (2)
O4—Mn1—N1	154.07 (6)	N3—C6—C8	132.4 (2)
O2—Mn1—N1	91.04 (6)	N3—C6—C3	105.50 (16)
O1—Mn1—N1	102.91 (6)	C8—C6—C3	122.1 (2)
N2—Mn1—N1	73.63 (6)	C10—C7—C3	116.5 (2)
C12—S1—C9	90.09 (11)	C10—C7—H7	121.7
C5—O1—Mn1	116.44 (11)	C3—C7—H7	121.7
C5^i^—O2—Mn1	116.44 (12)	C11—C8—C6	116.0 (2)
C1—O3—Mn1	114.82 (12)	C11—C8—H8	122.0
C1^ii^—O4—Mn1	114.64 (12)	C6—C8—H8	122.0
C12—N1—C4	110.24 (18)	C4—C9—S1	110.03 (17)
C12—N1—Mn1	135.57 (15)	C4—C9—H9	125.0
C4—N1—Mn1	113.89 (12)	S1—C9—H9	125.0
C2—N2—C3	105.18 (15)	C7—C10—C11	122.3 (2)
C2—N2—Mn1	116.34 (12)	C7—C10—H10	118.8
C3—N2—Mn1	138.48 (13)	C11—C10—H10	118.8
C2—N3—C6	107.05 (16)	C8—C11—C10	122.2 (2)
C2—N3—H3	126.5	C8—C11—H11	118.9
C6—N3—H3	126.5	C10—C11—H11	118.9
O4^ii^—C1—O3	126.52 (17)	N1—C12—S1	115.01 (18)
O4^ii^—C1—C1^ii^	116.9 (2)	N1—C12—H12	122.5
O3—C1—C1^ii^	116.54 (19)	S1—C12—H12	122.5
N2—C2—N3	112.80 (17)		

Symmetry codes: (i) −*x*+1, −*y*+2, −*z*+1; (ii) −*x*, −*y*+2, −*z*.

### Hydrogen-bond geometry (Å, º)

**Table:**

*D*—H···*A*	*D*—H	H···*A*	*D*···*A*	*D*—H···*A*
N3—H3···O1^iii^	0.86	1.96	2.812 (2)	170
C12—H12···O3^iv^	0.93	2.52	3.137 (3)	124

Symmetry codes: (iii) *x*, −*y*+3/2, *z*−1/2; (iv) −*x*+1, −*y*+2, −*z*.
